# Venetoclax Initiation in Chronic Lymphocytic Leukemia: International Insights and Innovative Approaches for Optimal Patient Care

**DOI:** 10.3390/cancers16050980

**Published:** 2024-02-28

**Authors:** Mary Ann Anderson, Renata Walewska, Fidelma Hackett, Arnon P. Kater, Josie Montegaard, Susan O’Brien, John F. Seymour, Matthew Smith, Stephan Stilgenbauer, Ashley Whitechurch, Jennifer R. Brown

**Affiliations:** 1 Department of Clinical Haematology, Peter MacCallum Cancer Centre and The Royal Melbourne Hospital, Melbourne, VIC 3000, Australia; ashley.whitechurch@petermac.org; 2 Division of Blood Cells and Blood Cancers, The Walter and Eliza Hall Institute, Melbourne, VIC 3000, Australia; 3 Peter MacCallum Cancer Centre, Royal Melbourne Hospital, University of Melbourne, Melbourne, VIC 3000, Australia; 4 University Hospitals Dorset, NHS Foundation Trust, Bournemouth BH7 7DW, UK; renata.walewska@uhd.nhs.uk; 5 Cancer Services Directorate, University Hospital Limerick UL Hospitals Group, St. Nessan’s Road, V94 F858 Limerick, Ireland; fidelma.hackett@hse.ie; 6 Department of Hematology, Cancer Center Amsterdam, Amsterdam University Medical Centers, 1081 HV Amsterdam, The Netherlands; a.p.kater@amsterdamumc.nl; 7 Dana-Farber Cancer Institute, Harvard Medical School, Boston, MA 02215, USA; josie_montegaard@dfci.harvard.edu (J.M.); jennifer_brown@dfci.harvard.edu (J.R.B.); 8 Chao Family Comprehensive Cancer Center, University of California Irvine, Orange, CA 92868, USA; obrien@hs.uci.edu; 9 Department of Haematology, Chesterfield Royal Hospital NHS Foundation Trust, Chesterfield S44 5BL, UK; matthew.smith59@nhs.net; 10 Division of CLL, Department of Internal Medicine III, Ulm University, 89081 Ulm, Germany; stephan.stilgenbauer@uniklinik-ulm.de

**Keywords:** chronic lymphocytic leukemia, multidisciplinary, obinutuzumab, onboarding, ramp-up, rituximab, tumor lysis syndrome, venetoclax

## Abstract

**Simple Summary:**

Venetoclax has proven a viable option for treatment of chronic lymphocytic leukemia (CLL), with high response rates and a generally manageable safety profile. Management considerations associated with venetoclax initiation include the risk of tumor lysis syndrome (TLS), which requires close attention and prompt management. Administration of venetoclax in a safe manner through a slow ramping up of the dose over a 5-week period, along with proper assessment, preparation, and initiation, are essential and have been successful in reducing the risk of TLS in patients with CLL. This review summarizes hypothetical patient case scenarios and emphasizes the importance of a collaborative team effort, with perspectives from highly respected clinicians in the field offering invaluable insight for optimal patient care and treatment strategies.

**Abstract:**

Venetoclax, a highly selective, oral B-cell lymphoma 2 inhibitor, provides a robust targeted-therapy option for the treatment of chronic lymphocytic leukemia (CLL), including patients with high-risk del(17p)/mutated-*TP53* and immunoglobulin heavy variable region unmutated CLL and those refractory to chemoimmunotherapy across all age groups. Due to the potent pro-apoptotic effect of venetoclax, treatment initiation carries a risk of tumor lysis syndrome (TLS). Prompt and appropriate management is needed to limit clinical TLS, which may entail serious adverse events and death. Venetoclax ramp-up involves gradual, stepwise increases in daily venetoclax dosing from 20 mg to 400 mg (target dose) over 5 weeks; adherence to on-label scheduling provides a tumor debulking phase, reducing the risk of TLS. The key components of safe venetoclax therapy involve assessment (radiographic evaluation and baseline blood chemistry), preparation (adequate hydration), and initiation (blood chemistry monitoring). In addition to summarizing the evidence for venetoclax’s efficacy and safety, this review uses hypothetical patient scenarios based on risk level for TLS (high, medium, low) to share the authors’ clinical experience with venetoclax initiation and present global approaches utilized in various treatment settings. These hypothetical scenarios highlight the importance of a multidisciplinary approach and shared decision-making, outlining best practices for venetoclax initiation and overall optimal treatment strategies in patients with CLL.

## 1. Introduction 

The highly selective, oral B-cell lymphoma 2 (BCL-2) inhibitor venetoclax has been shown to be very effective for the treatment of chronic lymphocytic leukemia (CLL) [1]. BCL-2 is highly expressed in CLL cells, and venetoclax blocks antiapoptotic BCL-2 signaling by functioning as a BCL-2 homology domain 3 (BH3) mimetic. Treatment of CLL cells with venetoclax induces the rapid onset of apoptosis in vitro and in vivo via a TP53-independent mechanism [2,3], providing the rationale for evaluating use of venetoclax as targeted therapy in CLL. In clinical trials, the efficacy and safety of venetoclax was demonstrated in both relapsed/refractory (R/R) disease and in the first-line setting. Venetoclax was initially granted accelerated approval by the United States Food and Drug Administration (FDA) in 2016 as continuous monotherapy for treatment of R/R CLL with del (17p) [4,5,6]. The label has since expanded, with venetoclax now approved by many international agencies including the FDA, European Medicines Agency (EMA), and other regulatory agencies as fixed-duration combination therapy with either rituximab (for R/R CLL) or obinutuzumab (for treatment-naïve disease). Venetoclax was recently approved by the EMA in combination with ibrutinib (for treatment-naïve disease) [7,8,9]. Fixed-duration therapy with venetoclax is an effective alternative to continuous treatment approaches with Bruton tyrosine kinase (BTK) inhibitors [10]. The choice of venetoclax-based therapy versus other targeted agents and chemoimmunotherapy depends on multiple factors, including patient age and comorbidities, *TP53* status, immunoglobulin heavy variable region (*IGHV*) mutational status, goals of treatment, tumor bulk, cardiac history and status, renal function, and concomitant medications [10,11,12]. Prior therapy (e.g., chemotherapy, BCL-2 inhibitor, BTK inhibitor) and response depth and duration also affect treatment decisions for patients with R/R CLL [10].

Owing to its potent pro-apoptotic effect on malignant cells, venetoclax poses a risk of inducing tumor lysis syndrome (TLS) [13]. TLS risk is evaluated before initiating venetoclax, and the determined risk level dictates prophylaxis and administration procedures as per the product information to ensure safe onboarding of this therapy for patients with CLL [7]. A gradual, 5-week venetoclax ramp-up with appropriate debulking, prophylaxis, and monitoring has been shown to be safe and effective, and it reduces the risk of TLS and the need for related hospitalization [14]. However, this regimen can be challenging to implement in many health care settings, and practitioners are devising real-world solutions to safely onboard patients with venetoclax—examples of which we will highlight in this review. 

In a review of the literature (January 2010–June 2021), real-world studies reported a significant reduction in TLS incidence when debulking strategies were used in patients with intermediate or high risk of TLS (using venetoclax monotherapy or venetoclax combination regimens) [14]. Some variability in reported rates of biochemical changes may stem from a failure to strictly adhere to ramp-up procedures specified in the product labeling, as better adherence to recommended protocols can mitigate TLS risk. Since the initial accelerated approval of venetoclax in 2016, healthcare providers have accrued a wealth of experience with this agent and have developed approaches to implement on-label initiation safely and effectively. This review draws on published data as well as the authors’ clinical experience with venetoclax to outline best practices for initiation in patients with CLL. Additionally, this review describes hypothetical patient scenarios to illustrate global approaches used in various treatment settings and emphasizes the importance of a multidisciplinary approach and shared decision-making, with the overall goal of equipping healthcare providers to deliver an optimal experience for patients undergoing treatment with venetoclax.

## 2. Clinical Trials and Real-World Studies of Venetoclax in Patients with CLL

### 2.1. Efficacy

Results from M13-982 (NCT01889186), a phase 2 trial of venetoclax monotherapy (N = 107) in patients with R/R CLL and del(17p), showed an overall response rate of 79.4% [5,6], leading to the FDA’s accelerated approval of venetoclax in the segment of the patient population with at least 1 prior therapy [15]. In a randomized phase 3 MURANO trial (NCT02005471; N = 389), the combination of venetoclax plus rituximab (for a fixed duration of 2 years) resulted in a response rate of more than 90% and a significant improvement in progression-free survival (PFS) for patients with R/R CLL, reducing the risk of disease progression or death by 81% compared to bendamustine plus rituximab [16]. This resulted in the FDA and EMA approvals of venetoclax plus rituximab in patients with R/R CLL [7,8]. These survival benefits of venetoclax were sustained 3 years after treatment cessation (5-year follow-up), with a median PFS of 53.6 months (venetoclax plus rituximab) compared with 17.0 months (bendamustine plus rituximab; *p* < 0.0001) [17]. Compared to the MURANO trial, a real-world analysis from the Polish Adult Leukemia Study group in patients with very high-risk R/R CLL receiving the combination of venetoclax plus rituximab, yielded a shorter median PFS (36.97 months [95% CI 24.5, not reached]) [18]. The randomized phase 3 CLL14 study (NCT02242942; N = 432) showed a significant decrease in disease progression or death (hazard ratio [HR], 0.35 [*p* < 0.001]) and longer PFS at 24-months (88.2% vs. 64.1%) after fixed-duration venetoclax plus obinutuzumab versus chlorambucil plus obinutuzumab in patients with previously untreated CLL and coexisting conditions [19], leading to FDA and EMA approvals of venetoclax plus obinutuzumab in first-line therapy [7,8]. At 5-year follow-up, PFS remained superior (HR, 0.35 [*p* < 0.0001]) with a higher PFS rate in the venetoclax plus obinutuzumab group (62.6%) compared to chlorambucil plus obinutuzumab (27.7%) and no significant difference noted in overall survival (OS), although at the end of treatment an undetectable minimal residual disease status with a cutoff at 10^−4^ was associated with longer OS [20]. Additionally, the re-emergence of detectable disease was significantly slower following venetoclax plus obinutuzumab compared with chlorambucil plus obinutuzumab [21]. Results from GLOW (NCT03462719), a phase 3 trial (N = 211) in older patients and/or those with comorbidities with previously untreated CLL, showed that the ibrutinib (3-cycle lead in) plus venetoclax regimen resulted in a significantly longer PFS compared to chlorambucil plus obinutuzumab (HR, 0.216; *p* < 0.001) [9]. This combination is approved by the EMA for adult patients with previously untreated CLL [22]. In the CAPTIVATE (NCT02910583) phase 2 trial, fixed-duration ibrutinib (3-cycle lead in) plus venetoclax resulted in a complete response rate of 56% in patients aged ≤70 years with previously untreated CLL (Table 1) [23]. In a phase 3 randomized study (NCT0290051), the combination of venetoclax with obinutuzumab or ibrutinib resulted in a significantly higher number of patients with undetectable minimum residual disease at 15 months (venetoclax- obinutuzumab [86.5%]; venetoclax-obinutuzumab-ibrutinib [92.2%] vs. chemoimmunotherapy [52.2%]; *p* < 0.001 for both comparisons). In addition, at 3 years, the median PFS was 87.7% (HR, 0.42) in the venetoclax-obinutuzumab group, 90.5% (HR, 0.32) in the venetoclax-obinituzumab-ibrutinib group, but only 75.5% following the chemotherapy regimen (*p* < 0.001 for both comparisons) [24].

In large, real-world studies, high response rates were observed in patients with either R/R or treatment-naïve CLL [25,26,27,28,29,30]. More specifically, most studies reported response rates between 80%–90% [25,26,27], with 2-year best overall response rates of more than 90% in patients who received venetoclax as a monotherapy or in combination with rituximab (Table 2) [28]. 

### 2.2. Safety

Adverse events with venetoclax generally are considered tolerable and manageable. In key trials of patients with CLL with del(17p) and who were treatment-naïve or had R/R disease, neutropenia was the most common grade ≥ 3 adverse event (38–58%) among others, including thrombocytopenia, infections, anemia, and infusion-related reaction (IRR; Table 1) [6,16,17,19,20]. In an integrated safety analysis of venetoclax in patients with R/R CLL based on three phase 1/2 trials using continuous single-agent therapy (N = 350) [31], the most common adverse events of any grade were diarrhea, neutropenia, nausea, anemia, fatigue, and upper respiratory tract infections. Discontinuations due to adverse events occurred in 10% of patients, and 8% of patients died, mainly due to disease progression [31]. In a retrospective analysis of venetoclax-related adverse events for patients with CLL (N = 297), the most common grade 3 or 4 adverse events were neutropenia, thrombocytopenia, infection, febrile neutropenia, and diarrhea (Table 2) [29]. Dose reductions were required in 29% of patients, dose interruptions in 32% of patients, and discontinuations due to adverse events in 7.4% of patients [29]. 

A retrospective analysis of the MURANO study assessed the impact of early venetoclax discontinuation or dose reduction [32]. Treatment interruption due to adverse events occurred in 69% of patients and was most often due to neutropenia; dose reductions were required in 23% of patients, but these did not adversely impact PFS. Only premature discontinuation affected outcomes, suggesting prompt, effective management of venetoclax-related adverse events enables resumption of therapy and the maintenance of efficacy [32]. 

In a real-world pharmacovigilance study of venetoclax-related adverse events in patients with CLL or other hematologic malignancies using the FDA Adverse Event Reporting System database (19,107 cases of adverse events) [33], the median time to occurrence of adverse events was 31 days (range, 7–131 days). Half of the events occurred in the first 30 days and approximately 70% in the first 3 months. Neutropenia was common (40–50%), and the occurrence of grade ≥ 3 neutropenia was associated with risk of pneumonia. Infections accounted for nearly a quarter of venetoclax-related adverse events (pneumonia was the most common), which is consistent with the immunosuppression associated with the underlying CLL [33]. Most adverse events can be managed with additional supporting medication (e.g., nausea with ondansetron or prochlorperazine; diarrhea with loperamide). However, careful monitoring for infections and judicious use of venetoclax in patients with pre-existing infections is warranted.

Combining venetoclax with antibody therapy or other targeted agents, such as rituximab and BTK inhibitors, may increase the risk of adverse reactions. More specifically, IRRs are frequent when venetoclax is used with obinutuzumab, particularly during the first obinutuzumab dose [19]. To reduce risk, patients should be premedicated with corticosteroids, antipyretics, and diphenhydramine the night prior to, the morning of, and immediately prior to infusion initiation, with an initial fractionated dose of obinutuzumab administered on the first day and a very slow infusion rate of obinutuzumab; both can be increased subsequently if no IRRs occur [7]. Other obinutuzumab-related adverse events such as neutropenia, thrombocytopenia, and infection can occur. Hepatitis B reactivation and progressive multifocal leukoencephalopathy have also been reported [7], but are rare events; hepatitis B should be tested for prior to therapy. In the phase 3 MURANO study, the most common adverse event of any grade in the venetoclax plus rituximab group was neutropenia, followed by infections and infestations [16].

### 2.3. Risk of Tumor Lysis Syndrome

Because venetoclax treatment poses a risk of TLS, great care is required to prevent and manage TLS, especially in patients at high risk. If not managed promptly and appropriately, laboratory TLS (laboratory evidence of metabolic changes, without symptoms such as hyperuricaemia, hyperkalaemia, hyperphosphataemia, secondary hypocalcaemia and uraemia) can worsen to clinical TLS (defined as laboratory TLS with clinical consequences such as acute renal failure, cardiac arrhythmias, seizures, and death [34]). Any evidence of laboratory TLS requires immediate action such as withholding the next day’s dose of venetoclax and reassessing after 24–48 h; then resuming at the same dose if the laboratory TLS is resolved and at a reduced dose if the clinical TLS is resolved [34]. The importance of adhering to venetoclax administration guidelines has been illustrated by a pooled analysis of venetoclax clinical trials and post-marketing studies (N = 1138) [13]. When recommended mitigation measures were followed, the overall TLS incidence was 1.8% (laboratory TLS 1.8% [clinical TLS, <1%]), with all patients resuming therapy after transient TLS, and no irreversible sequelae [13]. Key potential causes of TLS were suboptimal blood chemistry monitoring, inappropriate venetoclax dose modification for drug-drug interactions, underestimating the TLS risk level, underestimating the degree of renal dysfunction, failure to adhere to the recommended ramp-up protocol, and failure to follow hydration guidelines [13]. 

In the MURANO trial (NCT02005471), grade 3/4 TLS was reported in 6/194 patients (3.1%) in the venetoclax-rituximab group and in 2/188 patients (1.1%) in the bendamustine-rituximab group; clinical TLS was reported in 1 patient in each treatment group [16]. In the phase 3 GLOW study (NCT03462719), no cases of TLS were reported in the ibrutinib-venetoclax group. TLS was reported in 5.7% of the chlorambucil-obinutuzumab group [9]; these cases were likely attributable to the obinutuzumab. In the phase 3 CLL14 study (NCT02242942), TLS was reported in 1.4% of patients in the venetoclax-obinutuzumab group (all cases occurred prior to venetoclax treatment) and 2.3% of patients in the chlorambucil-obinutuzumab group; none of the cases was defined as clinical TLS in either group [19]. 

In a subset of patients for whom the 5-week ramp-up procedure was followed, the incidence of TLS with venetoclax monotherapy was 1.2% (all laboratory TLS; no TLS-related deaths) [31]. Notably, obinutuzumab administered as a monotherapy [35], or in combination regimens without venetoclax, has been shown to induce TLS. In the pivotal phase 3 CLL11 study (NCT01010061), 4% of patients treated with obinutuzumab plus chlorambucil experienced TLS [36]. In an aggregated real-world cohort treated with venetoclax, clinical and laboratory TLS occurred in 2.7% and 5.7% of patients, respectively, and 1 TLS-related death occurred [29]. TLS risk could be predicted by pre-treatment TLS risk assessment and a creatinine clearance (CrCl) of < 80 mL/min [29]. The occurrence of TLS was dependent on tumor size, the presence of comorbidities (especially impaired renal function), and the venetoclax dose [33]. Obinutuzumab, when used as a pre-induction therapy in cycle 1 days 1 and 2, may reduce the risk of TLS if administered before venetoclax [19,37,38].

### 2.4. On-Label Venetoclax Initiation

Venetoclax ramp-up is designed to reduce the tumor burden gradually (e.g., debulk) by slowly increasing the daily dose to reach the targeted dosing of 400 mg, thus reducing the risk of TLS. Ensuring appropriate prophylaxis, including adequate hydration and anti-hyperuricemia drugs as indicated, can lower TLS risk [7,39]. An extended ramp-up schedule has been associated with a measured reduction in peripheral blood lymphocyte count, suggesting a more controlled cytotoxic effect compared to dose escalation [39]. Strict adherence to venetoclax on-label ramp-up guidelines results in low rates of TLS, which supports the assertion that treatment can be safely administered using this schedule. 

Key components of safe venetoclax therapy involve assessment, preparation, and initiation. An assessment of tumor burden (by radiographic evaluation of maximal nodal diameter with reevaluation during ramp-up), and a baseline blood chemistry test are recommended before venetoclax initiation to determine the risk of TLS. The risk level is evaluated based on multiple factors, including reduced renal function (CrCl < 80 mL/min per the label), tumor burden, and the presence of splenomegaly [7]. The tumor burden is categorized as low (all lymph nodes < 5 cm, and absolute lymphocyte count < 25 × 10^9^/L), medium (any lymph node 5 to <10 cm, or absolute lymphocyte count ≥ 25 × 10^9^/L), or high (spleen > 6 cm below costal margin, any lymph node ≥ 10 cm, or any lymph node ≥ 5 cm and absolute lymphocyte count ≥ 25 × 10^9^/L) [40,41]. Certain comorbidities, such as impaired renal function, may also increase the risk of TLS. Errors in lymph node classification (e.g., due to failure to image or inaccurate interpretation of scans from computed tomography) can result in an inaccurate determination of tumor burden and thus affect the determination of TLS risk and lead to inappropriate management. If a patient receives obinutuzumab prior to venetoclax, imaging should be repeated immediately prior to venetoclax initiation, to ensure a correct assessment of the risk of TLS. In addition, it has been shown that 2 cycles of obinutuzumab prior to initiation of venetoclax was an effective debulking strategy (success rate over 98%) for patients with absolute lymphocyte count > 25 × 10^9^/L and lymph nodes < 5 cm (medium risk TLS) [42]. 

Preparation consists of ensuring adequate hydration, orally and with an anti-hyperuricemic such as allopurinol. For those with a prior allopurinol allergy, febuxostat is a safe alternative agent [43]. Intravenous hydration during an outpatient stay may be considered for patients with a medium tumor burden and should be administered to all patients with a high tumor burden, possibly with rasburicase if the uric acid level is >8 mg/dL [44]. Prior to the administration of rasburicase, glucose-6-phosphate dehydrogenase deficiency may be excluded based on geographical region, as it can lead to methemoglobinemia in susceptible individuals [44]. In some institutions, this is routinely established during preparation for venetoclax ramp-up in all patients to avoid the need for rapid testing if rasburicase is urgently needed to treat uncontrolled hyperuricemia. In the case of a high tumor burden and following treatment with rasburicase, patients should start receiving oral allopurinol [45] 48–72 h postdose and continue until at least day 7 post final dose escalation. For low and medium risk cases of TLS, allopurinol should be started 48–72 h prior to the first dose of venetoclax [34]. Oral hydration should be initiated ≥ 2 days before the first dose and continued throughout ramp-up. Hydration is especially important on the first day of each dose escalation; however, older patients may struggle to drink enough water (1.5–2.0 L/day) or may have fluid restrictions due to heart failure and may require IV hydration under collaborative management with the heart failure care team [7].

Initiation of venetoclax dosing requires the monitoring of blood chemistry (potassium, uric acid, phosphorus, creatinine, lactate dehydrogenase, and calcium) around the first dose at each new level. Hospitalization can be considered for patients with a medium tumor burden and CrCl < 80 mL/min during administration of the initial 20-mg and 50-mg venetoclax doses. Hospitalization during administration of the initial 20-mg and 50-mg venetoclax doses is indicated for all patients with a high tumor burden [7]. Venetoclax dose modifications may be required for patients who develop specific grade 3/4 adverse events or have changes in blood chemistry or symptoms suggestive of TLS. Grade 3/4 neutropenia may require dose interruption/reduction, but usually dosing would be continued unaltered for asymptomatic grade 3 neutropenia. Treatment with growth factor support can help maintain absolute neutrophil count during ramp-up and the first several cycles of venetoclax, thus maintaining scheduled dosing [10]. Short-acting growth factor and/or intermittent dosing (e.g., once to twice per week) is usually adequate, although long-acting growth factor (e.g., pegfilgrastim) may have prolonged benefit for more than 4–6 weeks. Patients should avoid concomitant use of venetoclax with strong or moderate cytochrome P450, family 3, subfamily A (CYP3A) inhibitors (e.g., ketoconazole, itraconazole, fluconazole) or P-gp inhibitors (e.g., amiodarone, ketoconazole) [7,41] at initiation and during ramp-up because these agents alter venetoclax pharmacokinetics and increase the risk of TLS. Venetoclax dose reductions are required if these agents are unavoidable after the ramp-up period [7,41].

## 3. International Insights and Innovative Approaches Illustrated by Hypothetical Patient Scenarios

### 3.1. Patient Case 1—High Risk for TLS and Renal Failure 

**Figure 1:**
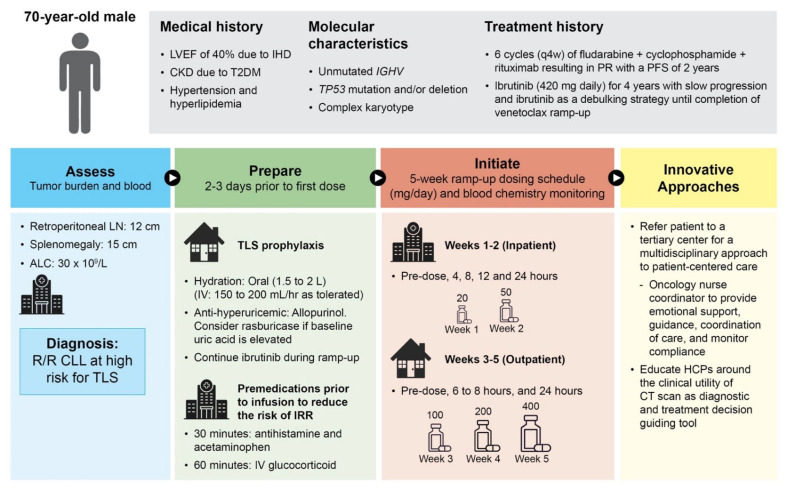
Patient case 1: High risk for tumor lysis syndrome (relapsed patient treated with continuous venetoclax). ALC, absolute lymphocyte count; CKD, chronic kidney disease; CLL, chronic lymphocytic leukemia; CT, computerized tomography; HCP, healthcare provider; IGHV, immunoglobulin heavy variable; IHD, ischemic heart disease; IV, intravenous; IRR, infusion-related reaction; L, liter; LN, lymph node; LVEF, left ventricular ejection fraction; mg, milligram; PFS, progression-free survival; PR, partial response; q4w, every 4 weeks; R/R, relapsed/refractory; T2DM, type 2 diabetes mellitus; TLS, tumor lysis syndrome; TP53, tumor protein 53.

A 70-year-old man with CLL has an extensive medical history that includes ischemic heart disease resulting in an impaired left ventricular ejection fraction of 40%, as well as chronic kidney disease (Crockoff Gault glomerular filtration rate of 30 mL/minute) due to type 2 diabetes (Figure 1). Other medical issues include hypertension and hyperlipidemia. A current molecular and cytogenetic workup revealed an unmutated *IGHV* gene status, the presence of a *TP53* mutation, and a complex karyotype [46]. This patient was heavily pre-treated and had received 6 cycles of fludarabine plus cyclophosphamide plus rituximab (prior to approval of targeted agents), which resulted in a partial response with a PFS of 2 years. Subsequently, continuous ibrutinib (420 mg daily) was administered to the patient, which resulted in PFS for 4 years followed by slow disease progression on this BTK inhibitor. At this point, the patient had R/R CLL with a high tumor burden due to a retroperitoneal conglomerate lymph node mass of 12 cm, a splenomegaly of 15 cm, and an absolute lymphocyte count of 80 × 10^9^/L; non-contrast radiographic evaluation was used due to the patient’s increased risk of renal damage. As it is recommended to continue on the BTK inhibitor during the transition period to the next line of therapy, especially through venetoclax ramp-up [47], he remained on ibrutinib until the completion of venetoclax ramp-up and the start of continuous venetoclax monotherapy. Before the initiation of continuous venetoclax treatment, the patient received increasing daily doses (20, 50, 100, 200, and 400 mg) of venetoclax every week over a 5-week period (Weeks 1 and 2 were inpatient; Weeks 3, 4, and 5 were at the treatment center as outpatient).

Because this hypothetical patient is at high risk of TLS, a more intensive intervention and specialized attention is required with close monitoring of renal function, serial tumor burden, and splenomegaly reduction. Appropriate prophylaxis with adequate hydration (150–200 mL/hr as tolerated prior to first dose with close fluid monitoring [i.e., twice daily weight, urine volume measuring] and diuretic supportive measures to avoid complications such as pulmonary edema), and administration of anti-hyperuricemics/rasburicase at the physician’s discretion is critical, along with monitoring and promptly addressing any emerging biochemical laboratory abnormalities, in particular hyperkalemia [34]. If available, the utilization of a nurse specialist is essential for continued patient education, the promotion of the patient’s adherence to the dosing schedule, the monitoring of blood chemistries, hydration, and supportive medications. 

### 3.2. Patient Case 2—Medium Risk for TLS with Potential Drug-to-Drug Interactions and Infusion Reactions

**Figure 2:**
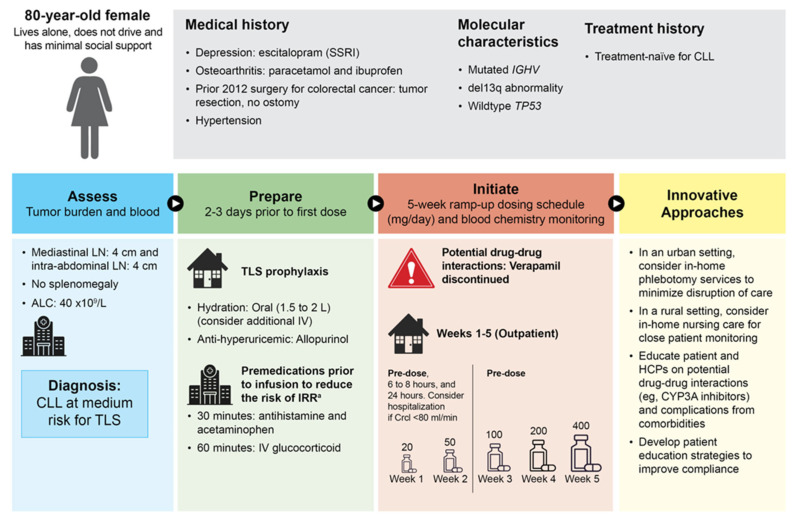
Patient case 2: medium risk for tumor lysis syndrome (treatment-naïve patient treated with fixed-duration venetoclax + obinutuzumab). ^a^ Some institutions will give corticosteroids (e.g., dexamethasone, 20 mg) and antihistamines (e.g., diphenhydramine, 25 mg) the night before and morning of the first obinutuzumab infusion, in addition to giving premedications right before the infusion. ALC, absolute lymphocyte count; CLL, chronic lymphocytic leukemia; CrCl, creatinine clearance; CT, computerized tomography; CYP3A, cytochrome P4503A; HCP, healthcare provider; IGHV, immunoglobulin heavy variable; IV, intravenous; IRR, infusion-related reaction; L, liter; LN, lymph node; mg, milligram; SSRI, selective serotonin reuptake inhibitor; TLS, tumor lysis syndrome.

An older woman aged 80 years was diagnosed with CLL (Figure 2). The patient has an extensive medical history which included depression treated with a serotonin selective reuptake inhibitor (i.e., escitalopram). Osteoarthritis was being treated with paracetamol and ibuprofen. In addition, she underwent tumor resection in 2012 for the treatment of colorectal cancer; no ostomy was required. Molecular testing showed a mutated *IGHV* status and a del(13q) abnormality by fluorescent in-situ hybridization; a *TP53* assessment did not show any deletion or mutation. For CLL, there was a medium tumor burden due to mediastinal nodes and intrabdominal nodes (both 4 cm). No splenomegaly was present, the lymphocyte count was 40 × 10^9^/L, the hemoglobin was 95 g/L, and the platelet count was < 100 × 10^9^/L. The patient had not received prior treatment for CLL. For hypertension, this patient had taken verapamil (moderate CYP3A inhibitor), which can interact with venetoclax and is contra-indicated during ramp-up. Verapamil may need to be halted before obinutuzumab infusion to avoid the risk of an increased severity of hypotension with any IRR. Co-administration of verapamil and venetoclax can significantly increase blood levels of venetoclax and increase the risk of TLS and is thereby contra-indicated during ramp-up, so it will need to be stopped. Of note, this patient lives alone, does not drive, and has minimal social support, which could present issues with adherence and/or the ability to attend appointments. 

This patient will receive the planned fixed-duration treatment with venetoclax plus Obinutuzumab, as the efficacy of this combination is high in patients with this genetic profile. Because the patient will receive obinutuzumab, proper premedications are required to reduce the risk of IRR. The drug regimens and protocols may differ between institutions. Some institutions will give corticosteroids (e.g., dexamethasone, 20 mg) and antihistamines (e.g., diphenhydramine, 25 mg) the night before and the morning of the first obinutuzumab infusion, in addition to giving premedications right before the infusion. At specific hospitals in the United Kingdom, during cycle 1 only (28 days), oral antihistamine (chlorphenamine, 4 mg) and oral acetaminophen (paracetamol, 1 mg) treatments are completed 30 min before obinutuzumab. Additionally, slow intravenous glucocorticoid (dexamethasone, 20 mg) treatment is completed 60 min before intravenous obinutuzumab on Days 1 (100 mg) and 2 (900 mg). In some cases, the above premedications are administered at Days 8 and 15 of cycle 1 prior to a 1000 mg IV of obinutuzumab; however, this hypothetical patient did not receive dexamethasone on Days 8 and 15 because no IRRs were present after Days 1 and 2. Premedications should be reduced and discontinued early when deemed possible by the treating physician. Starting a few days prior to Day 1 of the first cycle, an anti-hyperuricemic (allopurinol, 300 mg orally once daily) is administered for 4 weeks and reviewed after the first cycle is complete. Depending on the institution, other medications that might be administered but are not required per the venetoclax label include an anti-viral (e.g., acyclovir, 400 mg orally twice daily) and an antiemetic metoclopramide (e.g., 10 mg orally 3 times per day as required). 

One treatment scenario for this patient involves an urban setting (e.g., Limerick, Ireland), where the older patient receives an in-home phlebotomy service that is standard for outpatients during the ramp-up period. Utilizing in-home phlebotomy services not only reduces the burden on the patient by minimizing trips to the clinic and the associated out of pocket costs (e.g., gas, parking), it also reduces patient volume in the clinic. The blood samples are taken in the home and delivered to the hospital laboratory. The laboratory values are expeditiously processed and reviewed with the clinician in real time, and the patient will be updated and given follow-up instructions by phone. This is coordinated by the nurse specialist team. A multidisciplinary team including advanced pharmacists is critical to closely monitor potential drug-drug interactions or complications and care for this patient, who has a long history of medical issues. In this case, verapamil should be avoided; alternative medications should be considered during the initiation and dose-titration phases of venetoclax treatment.

A second treatment scenario is in a rural setting (e.g., regional Australia). In regional and remote geographic areas, access to suitable health services may be limited. Similar to the urban setting, education of the patient and clinician is important, along with scheduling and coordinating laboratory workup examinations. Changes in laboratory values (i.e., uric acid, phosphorus, potassium, and calcium) can occur within hours of treatment initiation and thus requires prompt management. To facilitate treatment, patients may require referrals to specialist treatment centers, with appropriate sourcing of local accommodation or inpatient stays. In other cases, patient care may be transferred back to a local hospital for ongoing management and co-managed through telehealth appointments with the referring physician and expert. A multidisciplinary approach with advanced practice nursing or pharmacists can bridge the gap in locations where medical service is limited [48,49]. Defining a clear program of roles and responsibilities, establishing escalation pathways, and upskilling/education to outsourced departments (i.e., emergency, day units) is crucial to ensure patient safety. This approach can ensure appropriate prophylaxis and employ more intensive measures such as intravenous hydration if the patient cannot maintain an adequate level of oral hydration, (the recommended volume is 6–8 glasses of water, or 1.5–2 L per day), frequent monitoring of laboratory values (e.g., 6–8 h and 24 h after first dose of venetoclax), and/or potential hospitalization based on assessments (first dose and potential subsequent dose increases of venetoclax) [34]. 

### 3.3. Patient Case 3—Low Risk for TLS and Busy Working Patient 

**Figure 3:**
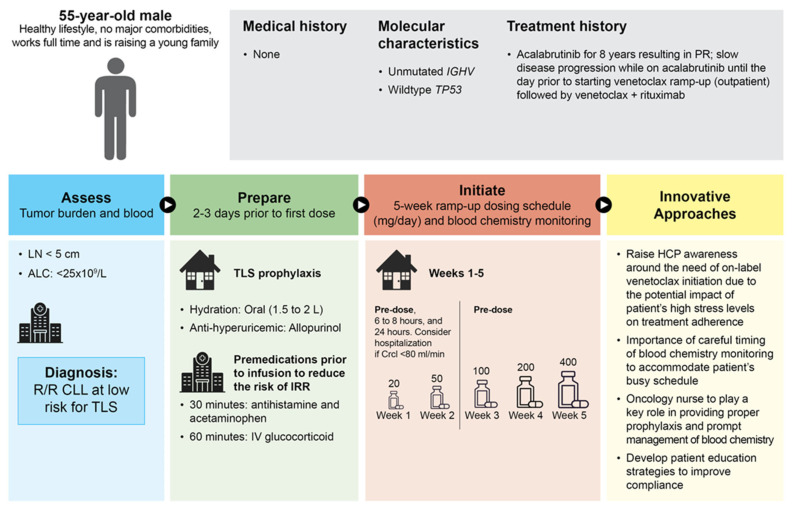
Patient case 3: low risk for tumor lysis syndrome (relapsed patient treated with continuous venetoclax monotherapy). ALC, absolute lymphocyte count; CLL, chronic lymphocytic leukemia; CrCl, creatinine clearance; HCP, healthcare provider; IGHV, immunoglobulin heavy variable; IRR, infusion-related reaction; IV, intravenous; L, liter; LN, lymph node; mg, milligram; PR, partial response; R/R, relapsed/refractory; TLS, tumor lysis syndrome.

A 55-year-old man maintains a healthy lifestyle, has no major comorbidities, works full time, and is raising a young family (Figure 3). Molecular testing showed an *IGHV* unmutated status and no *TP53* abnormalities. He previously received acalabrutinib for 8 years and achieved a partial response. However, CLL slowly progressed, with the next treatment option being ramp-up of continuous venetoclax monotherapy. For this patient, acalabrutinib was ceased the day before initiating venetoclax, although it should be noted that other patients may continue to receive acalabrutinib until the completion of the venetoclax ramp-up, particularly if the patient has aggressive disease progression and there are no issues accessing continued treatment. There was a low tumor burden because all lymph nodes were < 5 cm and the absolute lymphocyte count was < 25 × 10^9^/L. Certain challenges with this patient include work and family commitments, making it difficult to schedule treatment and/or appointments at a medical center. After the 5-week ramp-up treatment, the patient was scheduled to receive a combination of venetoclax plus rituximab.

To navigate these challenges, the clinician will need to be aware of and understand the necessity of on-label initiation and identify red flags regarding patient stress that pose potential risks to optimal treatment and drug adherence. Careful coordination of dose administration and laboratory tests to accommodate the patient’s busy schedule will be extremely important. In addition, the use of electronic tools and applications which contain calendars and reminders can be beneficial to keep track of all scheduled visits, dosing, and tests. For the venetoclax ramp-up, laboratory results will be obtained the day prior to dosing. On Day 1, two 10 mg tablets are taken orally, followed by laboratory tests performed at the medical center or office 6–8 h after the dose for rapid turnaround and action. On Day 2, morning bloodwork is to be completed on-site prior to dosing, with monitoring of potassium, calcium, phosphorus, and uric acid. On Day 7, pre-dose laboratory tests are performed prior to dosing. The same routine is followed for the second week, with the only difference being that the venetoclax dose is increased to 50 mg per day. For Weeks 3 and 4, the venetoclax dose is increased to 100 mg and 200 mg daily, respectively, along with the laboratory tests performed on Day 7 only [34]. For the first 2 weeks, during which more frequent blood work is collected (Supplementary Figure S1), the patient can be supported for 2–3 days per week to properly accommodate his schedule and ensure that he receives the necessary treatments and testing [34]. 

## 4. Patient Journey 

**Figure 4:**
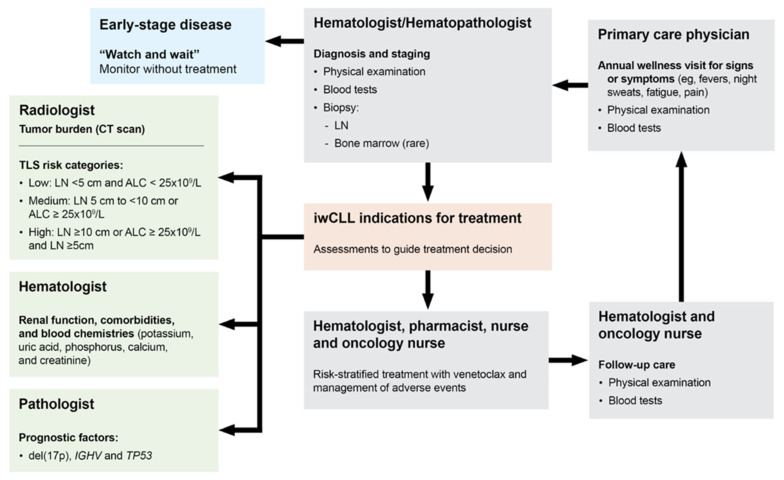
The journey of patients with CLL involves the coordination of a multidisciplinary team and shared decision-making. ALC, absolute lymphocyte count; CT, computerized tomography; del(17)p, deletion of chromosome 17p; IGHV, immunoglobulin heavy variable; iwCLL, International Workshop on Chronic Lymphocytic Leukemia; LN, lymph node; TLS, tumor lysis syndrome; TP53, tumor protein 53.

These cases highlight that a multidisciplinary team encompassing the patient, nurse practitioner/coordinator, hematologist, and pharmacist is essential for successful onboarding and facilitating personalized treatment plans [50], with the goal of achieving optimal patient care and treatment outcomes [51]. Care team members include primary care physicians, hematologists, pathologists, radiologists, nurses, and pharmacists, all of whom are responsible for different and overlapping roles such as proper diagnosis, (i.e., blood tests and physical examinations), risk assessments (i.e., staging), informative prognostics (e.g., mutational status) and/or therapy determination, and risk-stratified treatment and the management of adverse events (Figure 4). Certain factors and considerations for treatment are administration route(s), length of treatment (which can entail higher or lower costs [52]), insurance coverage, daily lifestyle, age, and potential side effects. It is important for patients to have a voice and share their preferences and concerns about various treatment options [53,54]. This will allow patients and providers to agree on a therapy that will fit both the clinical needs to effectively target CLL and the patient’s lifestyle for proper accommodation and convenience. Also of importance for treatment management is patient education and supplemental materials, such as diaries for fluid and medication record keeping and other support devices.

As described in the hypothetical patient cases, patients may need certain accommodations based on their age, location, level of support from family and society, and busy schedules, further emphasizing that numerous factors must be considered on a patient-by-patient basis. Shared decision-making is a continuous process that can change over time and requires constant communication, especially when patients notice changes or worsening of symptoms, regardless of whether they believe it is related to their current treatment regimen. 

## 5. Conclusions and Future Directions

Venetoclax provides a robust targeted-therapy option for the full spectrum of patients with CLL, including those with high-risk del(17p)/mutated-*TP53* CLL and those with disease refractory to chemoimmunotherapy. The initiation and ramp-up of venetoclax require proper assessment of the risk of TLS along with risk-stratified monitoring and mitigation measures to allow safe initiation and dose escalation. Venetoclax ramp-up is feasible in a variety of settings with sufficient personnel, on-site clinical laboratories, proper education, adequate planning, and relevant experience with venetoclax. Ambulatory care for moderate or high-risk TLS venetoclax dose escalations is present in many parts of the world and is becoming increasingly popular across specialist centers in the United Kingdom. These centers allow better coordination of logistics, scheduling, administration, and management of adverse events such as IRRs. In academic settings and large clinics, a dedicated nurse champion and/or patient coordinator can facilitate logistics pertaining to venetoclax administration, ramp-up, and the scheduling of patient visits and laboratory tests. Regimens, and specifically premedications as noted in the hypothetical medium-risk case for TLS, may vary based on the institution or practice, all with the goal of avoiding infusion reactions and minimizing the risk of adverse events. Alongside proper education, optimal patient-based treatment strategies can be achieved with a multidisciplinary care team and shared decision-making to navigate potential challenges in this continually evolving treatment landscape. This review summarized 3 hypothetical cases; however, depending on the specific factors described in each case, these treatment approaches can be applied to other cases in this patient population.

## Figures and Tables

**Table 1:** Summary of key venetoclax trials in patients with CLL.

Trial	Phase	N	Patients	Regimen ^a^	Efficacy ^b^	Most Common Grade ≥ 3 AEs	Grade ≥ 3 TLS ^c^
NCT01889186 [6] (M13-982)	2	107	R/R CLL with del(17p)	PO Ven step-up 20–400 mg qd over 4–5 wk; then PO Ven 400 mg qd to PD or D/C	ORR 79.4% CR/CRi 7.5% 18/45 (40%) uMRD 12-mo PFS 72.0%	Neutropenia 40.2% Anemia 17.8% Thrombocytopenia 15.0%	4.7% ^d^
NCT02005471 [16,17] (MURANO)	3	194 (Ven + R)	R/R CLL with 1–3 prior therapies	PO Ven step-up 20–400 mg qd over 5 wk; then IV R (375 mg/m^2^ C1D1, then 500 mg/m2 D1 C2–6) and 400 mg PO Ven for 2 yr, PD or unacceptable toxicity	ORR 92.3% CR/Cri 26.8% ^e^ 83.5% uMRD ^f^ mPFS 53.6 mo 5-yr OS 82.1%	Neutropenia 57.7% Infections/infestations 17.5% Anemia 10.8%	3.1% ^d^ < 1% ^fg^ [16]
		195 (BR)		IV B 70 mg/m^2^ D1, D2 for 6 cycles, and IV R (375 mg/m^2^ C1D1, then 500 mg/m2 D1 C2–6)	ORR 72.3% CR/Cri 8.2% ^e^ 23.1% uMRD ^f^ mPFS 17.0 mo 5-yr OS 62.2%	Neutropenia 38.8% Infections/infestation 21.8% Anemia 13.8%	1.1% ^d^ < 1% ^fg^ [16]
NCT02242942 [19,20,21] (CLL14)	3	216 (Ven + Obi)	Previously untreated CLL with CIRS ≥ 6	Obi IV D1 (100 mg C1D1, 900 mg C1D2, 1000 mg C1D8 and C1D15, then 1000 mg D1 of C2–6), and PO Ven on C1D22, 20–400 mg 5-week ramp-up, then 400 mg daily through C12	ORR 84.7% CR 49.5% mPFS NR 5-yr PFS 62.6% 5-yr OS 81.9% 4-yr MRD 18.1%	Neutropenia 52.8% Thrombocytopenia 13.7% IRR 9.0%	1.4% ^d,h,i^ [19]
		216 (Clb + Obi)		Clb 0.5 mg/kg D1 and D15 of C1–12, and PO Ven on C1D22, 20–400 mg 5-week ramp-up, then 400 mg daily through C12	ORR 71.3% CR 23.1% mPFS 36.4 mo 5-yr PFS 27.0% 5-yr OS 77.0% 4-yr MRD 1.9%	Neutropenia 48.1% Thrombocytopenia 15.0% IRR 10.3%	2.3% ^d,i^ [19]
NCT03462719 [9] (GLOW)	3	106 (Ibr + Ven)	Previously untreated CLL in older patients and/or those with comorbidities	3 cycles of Ibr lead-in at 420 mg once daily followed by 12 cycles of Ibr + Ven then Ven on C4, 20–400 mg 5-week ramp-up, then 400 mg daily on C5 onward	PFS HR 0.216; *p* < 0.001 ^f^ 24-mo PFS rate 84.4% 30-mo PFS rate 80.5% 55.7% best uMRD ^j^ 84.5% sustained uMRD ^k^ CR/CRi 38.7%	Neutropenia 34.9% Infections and infestations 17.0% Thrombocytopenia 5.7%	0
		105 (Clb + Obi)		Obi IV 1000 mg C1D1 (or 100 mg D1 and 900 mg D2), C2D8, and C1D15 and D2 of C2-C6 + Clb 0.5mg/kg on D1 and D15 and 15 of each cycle	24-mo PFS rate 44.1% 30-mo PFS rate 35.8% 21.0% best uMRD ^j^ 29.3% sustained uMRD ^j^ CR/CRi 11.4% ^e^	Neutropenia 49.5% Infections and infestations 10.5% Thrombocytopenia 20.0%	5.7%
NCT02910583 [23] (CAPTIVATE)	2	159	Previously untreated CLL	3 cycles of Ibr lead-in then 12 cycles of Ibr plus Ven (oral ibrutinib [420 mg/d]; oral venetoclax [5-week ramp-up to 400 mg/d]).	CR 55% uMRD 77% 2-year PFS 95% 2-year OS 98%	Neutropenia 33% Hypertension 6%	0

C, cycle; CIRS, Cumulative Illness Rating Scale; Clb, chlorambucil; CLL, chronic lymphocytic leukemia; CR, complete remission; CRi, complete remission with incomplete recovery of blood counts; D, day; D/C, discontinuation; Ibr, ibrutinib; IRR, infusion-related reaction; IV, intravenous; MRD, minimal residual disease; NR, not reached; Obi, obinutuzumab; ORR, overall response rate; OS, overall survival; PD, progressive disease; PFS, progression-free survival; PO, orally; qd, daily; R, rituximab; R/R, relapsed/refractory; TLS, tumor lysis syndrome; uMRD, undetectable minimal residual disease in blood and/or bone marrow; Ven, venetoclax. ^a^ All cycles were 28 days. ^b^ Assessed by independent review committee unless otherwise noted. ^c^ TLS was assessed by Howard’s criteria or the reference did not specify ^d^ TLS by laboratory measurement. ^e^ Investigator-assessed outcome. ^f^ At any time during the trial. ^g^ Clinical TLS. ^h^ All cases of TLS occurred before venetoclax treatment. ^i^ Did not meet the Howard criteria for clinical TLS. ^j^ In bone marrow at time of primary analysis by next generation sequencing. ^k^ In peripheral blood from 3 to 12 months after end of treatment.

**Table 2:** Summary of large, real-world studies of venetoclax trials in patients with CLL.

Reference	N	Countries	Patients	Regimen	Efficacy	Most Common Grade ≥ 3 AEs	TLS ^a^
Mato et al. [27]	270	US, UK	R/R CLL	Ven	ORR 81% CR 34% mPFS NR ^b^	Neutropenia 40.4% Thrombocytopenia 30.8% Neutropenic fever 8.6%	11.5%
	51			Ven + R or Obi	ORR 84% CR 32% mPFS NR ^b^	Neutropenia 34% Thrombocytopenia 23% Neutropenic fever 2.3%	5.8%
Roeker et al. [29]	297	US, UK	CLL (96% R/R)	Ven or Ven combo	N/A	Neutropenia 39.6% Thrombocytopenia 29.2% Febrile neutropenia 7.9%	2.7% ^c^ 5% ^d^
Zakeri et al. [30]	254	US	1L or 2L CLL	Ven-based	mTTNT-D NR ^e^ (Ven-Obi, Ven-R); mTTNT-D 13.5 mo ^d^ (Ven)	N/A	N/A
Herishanu et al. [26]	83	Israel	TN CLL	Ven-Obi or Ven-R	ORR 89.5% CR 68.4% uMRD: 12/14 (85.7%) 12-mo PFS 90.9%	Neutropenia 19.2% Infections 4.8% Febrile neutropenia 2.4%	1.2% ^c^
	116		R/R CLL	Ven-based	ORR 67.6% (BCRi-exposed); ORR 85.7% (BCRi-naïve) uMRD: 26/38 (68.4%) 12-mo PFS 81.1%	Neutropenia 17.2% Infections 21.5% Febrile neutropenia 2.6%	3.4% ^c^ 2.6% ^d^
Ysebaert et al. [28]	121	France	CLL	Ven	BORR (2 yr) 91.7% 2-yr PFS 71.7% 2-yr OS 79.6%	N/A	8.8%
	70		CLL	Ven + R	BORR (2 yr) 94.3% 2-yr PFS 77.9% 2-yr OS 80.6%	N/A	9.6%
Figueroa-Mora et al. [25]	170	UK	R/R CLL	Ven-based	ORR 85% CR/CRi 45.9%	N/A	1.1% ^c^ 2.4% ^d^

1L, first line; 2L, second line; AE, adverse event; BCRi, B-cell receptor inhibitor; BORR, best overall response rate; CLL, chronic lymphocytic leukemia; CR, complete remission; CRi, complete remission with incomplete recovery of blood counts; mTTNT-D, median time to next treatment or death; N/A, not available; NR, not reached; Obi, obinutuzumab; ORR, overall response rate; OS, overall survival; PFS, progression-free survival; R/R, relapsed/refractory; TLS, tumor lysis syndrome; TN, treatment naïve; UK, United Kingdom; uMRD, undetectable minimal residual disease in blood and/or bone marrow; US, United States; Ven, venetoclax. ^a^ TLS was assessed by Howard’s criteria or the reference did not specify ^b^ At 13.4-month median follow-up. ^c^ Clinical TLS. ^d^ TLS by laboratory measurement. ^e^ At 23.1-month median follow-up.

## Supplementary Materials

The following supporting information can be downloaded at: https://www.mdpi.com/article/10.3390/cancers16050980/s1, Supplementary Figure S1. Venetoclax ramp-up daily dosing and blood chemistry monitoring schedule^a^ [34] ^a^ Potassium, calcium, creatinine, phosphorus, uric acid (review in real time, evaluate and manage promptly).
