# Supplementary material for: Venetoclax Initiation in Chronic Lymphocytic Leukemia: International Insights and Innovative Approaches for Optimal Patient Care
Source: Cancers (Basel). 2024 Feb 28;16(5):980. doi: 10.3390/cancers16050980 (PMC10931175; doi:10.3390/cancers16050980)
Supplement: Supplementary file 1 [file cancers-16-00980-s001.zip › cancers-2782666-supplementary.pdf]

Supplementary Figure S1.

	DAY 1	DAY 2	DAY 3	DAY 4	DAY 5	DAY 6	DAY 7
**Week 1** **20 mg/day**	10 mg x 2<br>6-8 hour post-dose labs	10 mg x 2<br>24 hour post-dose labs	10 mg x 2	10 mg x 2	10 mg x 2	10 mg x 2	10 mg x 2<br>Pre-dose before next dose labs
**Week 2** **50 mg/day**	50 mg<br>6-8 hour post-dose labs	50 mg<br>24 hour post-dose labs	50 mg	50 mg	50 mg	50 mg	50 mg<br>Pre-dose before next dose labs
**Week 3** **100 mg/day**	100 mg	100 mg	100 mg	100 mg	100 mg	100 mg	100 mg<br>Pre-dose before next dose labs
**Week 4** **200 mg/day**	100 mg x 2	100 mg x 2	100 mg x 2	100 mg x 2	100 mg x 2	100 mg x 2	100 mg x 2<br>Pre-dose before next dose labs
**Week 5** **400 mg/day**	100 mg x 4	100 mg x 4	100 mg x 4	100 mg x 4	100 mg x 4	100 mg x 4	100 mg x 4

Supplementary Figure S1. Venetoclax ramp-up daily dosing and blood chemistry monitoring schedule[a] [34] [a]Potassium, calcium, creatinine, phosphorus, uric acid (review in real time, evaluate and manage promptly).
